# Placental determinants of fetal growth: identification of key factors in the insulin-like growth factor and cytokine systems using artificial neural networks

**DOI:** 10.1186/1471-2431-8-24

**Published:** 2008-06-17

**Authors:** Maria E Street, Enzo Grossi, Cecilia Volta, Elena Faleschini, Sergio Bernasconi

**Affiliations:** 1 Department of Pediatrics, University of Parma, 43100 Parma, Italy; 2 Centro Diagnostico Italiano, Via Saint Bon, Milan, Italy; 3 Department of Pediatrics, I.R.C.C.S "Burlo Garofalo", Trieste, Italy

## Abstract

**Background:**

Changes and relationships of components of the cytokine and IGF systems have been shown in placenta and cord serum of fetal growth restricted (FGR) compared with normal newborns (AGA). This study aimed to analyse a data set of clinical and biochemical data in FGR and AGA newborns to assess if a mathematical model existed and was capable of identifying these two different conditions in order to identify the variables which had a mathematically consistent biological relevance to fetal growth.

**Methods:**

Whole villous tissue was collected at birth from FGR (N = 20) and AGA neonates (N = 28). Total RNA was extracted, reverse transcribed and then real-time quantitative (TaqMan) RT-PCR was performed to quantify cDNA for IGF-I, IGF-II, IGFBP-1, IGFBP-2 and IL-6. The corresponding proteins with TNF-α in addition were assayed in placental lysates using specific kits. The data were analysed using Artificial Neural Networks (supervised networks), and principal component analysis and connectivity map.

**Results:**

The IGF system and IL-6 allowed to predict FGR in approximately 92% of the cases and AGA in 85% of the cases with a low number of errors. IGF-II, IGFBP-2, and IL-6 content in the placental lysates were the most important factors connected with FGR. The condition of being FGR was connected mainly with the IGF-II placental content, and the latter with IL-6 and IGFBP-2 concentrations in placental lysates.

**Conclusion:**

These results suggest that further research in humans should focus on these biochemical data. Furthermore, this study offered a critical revision of previous studies. The understanding of this system biology is relevant to the development of future therapeutical interventions possibly aiming at reducing IL-6 and IGFBP-2 concentrations preserving IGF bioactivity in both placenta and fetus.

## Background

Most cases of fetal growth restriction (FGR) are still of unknown origin (1). The interest in FGR has grown because approximately 13% of these subjects do not present a catch-up growth (2), and in recent years, the concept of a "Fetal Origin of Adult Disease" has been introduced to describe modifications in utero that might influence adult patho-physiology (3).

The IGF system is recognized to be crucial for fetal growth, as experiments in knockout mice have shown [4-7]. It is well known that IGF-I and IGF-II are both synthesised in the placenta [8-10]. IGFBP-1, IGFBP-2, IGFBP-4 and IGFBP-6 are expressed by all placenta cell types while IGFBP-3 and IGFBP-5 are expressed only by some [11]. We have recently shown that IGF-I, IGFBP-2 and IGF-II gene expression was increased in the placentas and that cord serum IGFBP-1 and IGFBP-2 were also increased in FGR [12].

Cytokines are thought to play an important role in regulating placenta development and growth although they are poorly studied. The placenta produces the pro-inflammatory cytokine IL-6, and TNF-α [13,14] and human decidua cells, in vitro, secrete IL-6 [15]. Very few fetal data have been published. Blood cultures stimulated with Lipopolysaccharide S, show an increase in placental IL-6, IL-1β, and TNF-α [16]. Recently, IL-6 gene expression and protein content in the placenta have been found significantly increased in FGR newborns and to be positively related with IGFBP-1 and IGFBP-2 gene expressions[12], confirming cytokine and IGF system relationships as previously described [17-20].

However, all above data are observational and conclusions come from traditional statistical analysis. In recent years a new array of technical tools and techniques has developed which has allowed a better understanding of biological networks. These advances have indicated that most networks in social, technological and biologic systems have common designs that are governed by simple and quantifiable organizing principles and that networks pervade all aspects of human health. Soon these advances will affect medical practice and a "network medicine" will develop [21].

The method used by Artificial Neural Networks (ANNS) aims to understand natural processes and recreate those processes using automatic models. These networks determine a noteworthy improvement, compared with traditional methods of analysis, and allow a method of forecasting with better and safer understanding of the relationships between variables, in particular non-linear relationships [22]. ANNs function by initially learning a known set of data from a given problem with a known solution (training) and then the networks, inspired by the analytical processes of the human brain, are able to reconstruct the imprecise rules which may be underlying a complex set of data (testing). In recent years ANNs have been used successfully in medicine [23-29], and have been applied to solve clinical problems in pediatrics including those related to preterm births [30-34]. Common algorithms of linear projections of variables as the Principal Component Analysis do not require any specific distribution of data. However, a limitation of these methods is that if the relationships between variables are non linear, it is not able to preserve, with adequate accuracy, the geometrical structure of the original space. Moreover, mapping is generally based on a specific kind of "distance" among variables and gives origin to a "static" projection of possible associations losing the intrinsic dynamics due to active interactions of variables in living systems of real world [35]. These limitations have been recently overcome using a new paradigm of variables mapping which creates a sort of semantic connectivity and has been described by Buscema and Grossi [35].

Using this latter method and supervised ANNS, we aimed to analyse a known set of clinical and biochemical data in FGR and normal newborns to establish if a mathematical model existed and was capable of identifying conditions of FGR and appropriateness for gestational age, identifying the variables, among those analysed, which had a mathematically consistent biological relevance to fetal growth, and their relationships. We showed that in particular, IGF-II, IGFBP-2 and IL-6 concentrations in placental lysates were the most important determinants of fetal growth.

## Methods

### Subjects

Twenty FGR and 28 appropriate for gestational age (AGA) births were followed. AGA births were defined on the basis of a normal birth weight (< 80th and > 10th centile) with respect to the Italian standards of referral [36] a normal pregnancy and the absence of maternal risk factors. All pregnancies were dated correctly by ultrasound during the first trimester of gestation. The FGR pregnancies were diagnosed by ultrasound according to the following criteria: abdominal circumference < 10th centile and shift of fetal growth with a reduction of abdominal circumference with respect to the measure taken within the 20th week of gestation. The diagnosis of FGR was made within the 32nd week of gestation and was ascribed to a probable placental cause after excluding other causes as infections, chromosomal abnormalities, genetic syndromes, maternal malnutrition, substance abuse, gross placental abnormalities and multiple fetuses.

No cases with increased blood pressure, gestational diabetes or reduced amount of amniotic fluid were included in the study. All neonates, both FGR and AGA, were delivered by elective caesarean section (CS) to avoid confounding factors in placental assays. The indications for CS in the AGA newborns were: intra-hepatic colestasis in a woman who had undergone a previous CS; premature rupture of membranes with breech presentation and delivery within 4 hr with no signs of infection; refusal of vaginal delivery for psychological reasons; and elective CS because of a previous CS.

At birth we collected the following information: maternal age, weight at birth of both parents, body mass index (BMI) of the mother before pregnancy and at delivery, previous gynaecological history, medical history during pregnancy, fetal biophysical data (exact duration of pregnancy, growth trend, fetal and maternal doppler velocimetry data in FGR, Non Stress Test), clinical data at delivery (indication for CS, neonatal sex, weight, length, head circumference, Apgar score, acid-base equilibrium, and perinatal data), and weight and macroscopic appearance of the placenta.

The main clinical data are reported in Table 1.

**Table 1:** Main features at birth of fetal growth restricted and appropriate for gestational age newborns

	**FGR**	**AGA**
**Males/Females**	9M/11F	15M/13F
**Gestational age (weeks)**	33.4 ± 0.8*	36.7 ± 0.5
**Weight of Placenta (gr)**	349.8 ± 35.3 *	615.9 ± 27.9
**Weight at birth (Kg)**	1.5 ± 0.1 *	2.9 ± 0.1
**Length at birth (cm)**	41.1 ± 1.3 *	49.7 ± 0.6
**Head circumference at birth (cm)**	29.4 ± 0.8 *	34.2 ± 0.4

FGR: fetal growth restricted; AGA: appropriate for gestational age: * p < 0.05 versus AGA

### Collection of biological material

In all cases, four fragments of perifunicular villous tissue of approximately 5 mm^3 ^were taken close to the fetal plate, rinsed repeatedly in sterile saline solution at 0°C. Storage conditions were standardized as previously described [12].

### Isolation of RNA

RNA extraction was performed as previously described [12].

### cDNA synthesis

Complementary DNA (cDNA) was synthesized using 1 μg of total RNA sample according to the recommendations of the manufacturer (Applied Biosystems, Foster City, California), and as previously described [12].

### TaqMan Assay on Demand Gene Expression

Applied Biosystems TaqMan Assay-on-Demand Gene Expression pre-designed primers and probes were used. To normalise for variables in gene expression, two control housekeeping genes were used, Ubiquitin (Assay ID: Hs00261902-m1) and 18S ribosomal RNA (Assay ID: Hs99999901-s1) [37]. IGF-I (Assay ID: Hs00153126-m1), IGF-II (Assay ID: Hs00171254-m1), IGFBP-1 (Assay ID: Hs00426258-m1), IGFBP-2 (Assay ID: Hs00167151-m1) and IL-6 (Assay ID: Hs00174131-m1) primers were used according to the manufacturer's instructions. Special attention was paid to selecting genes belonging to different functional classes, which significantly reduced the chance that genes might be co-regulated. Real-Time Quantitative RT-PCR was performed on a TaqMan ABI 7700 Sequence Detector System (Applied Biosystems) as previously described [12,37].

### Total protein content

The lysates were extracted as previously described [12]. The total protein content was expressed in μg per mg of placenta.

### Protein assays

Total IGF-I, IGF-II, IGFBP-2 and IL-6 were measured as previously described [12]. TNF-α was assayed using an ultrasensitive ELISA method ((Biosource International Camarillo, CA, USA), The sensitivity of the method was < 0.09 pg/ml, the intra- and inter-assay coefficients of variation were 6.7 and 7.7%, respectively.

### Database

We aimed to re-analyze from a completely new perspective most of the data, with TNF-α in addition, we obtained from our previous study, comparing FGR and AGA newborns [12]. Sixteen variables were selected for the analysis from the entire database for 48 subjects. The indexes of linear correlation among these variables were very low (data not shown).

Clinical, medical history and biochemical data were administered to the ANNS (Table 2). The models we used aimed at correct classification of the subjects in two classes: 1) FGR (fetal growth restriction), 2) AGA (appropriate for gestational age).

**Table 2:** List of variables analysed using Artificial Neural Networks (ANNS) and their linear correlation index (r^2^) with each target variable.

	**Variables**	**Correlation index **(r^2^)
1	Chronological age of the mother at delivery (years)	0,11
2	Sex of the newborn	0
3	Gestational age (weeks)	0,22
4	Other siblings (yes or No)	0,001
5	Number of other siblings	0,005
6	Number of previous abortions	0,000
7	Total placental protein content in lysates (mg/ml)	0,007
8	IGF-II concentration in placental lysates (ng/mg)	0,16
9	IGFBP-2 concentration in placental lysates (ng/mg)	0,05
10	IL-6 concentration in placental lysates (pg/mg)	0,09
11	TNF-α concentration in placental lysates (ng/mg)	0,05
12	IGF-I relative gene expression in placenta (A.U./18S, UBQ)	0,01
13	IGF-II relative gene expression in placenta (A.U./18S, UBQ)	0,02
14	IGFBP-1 relative gene expression in placenta (A.U./18S, UBQ)	0,05
15	IGFBP-2 relative gene expression in placenta (A.U./18S, UBQ)	0,08
16	IL-6 relative gene expression in placenta (A.U./18S, UBQ)	0,16

Biochemical data were measured as specified in materials and methods. Gene expressions are in arbitrary units (AU) with respect to two house-keeping genes, 18 S ribosomal RNA (18S) and ubiquitin (UBQ). Specific protein concentrations in placental lysates are ng or pg per mg of placenta.

### ANNs analysis

The ANNs model implemented to process the data in our study belonged to the Supervised networks in which the result of the processing (the desired output) is already defined. Supervised ANNs calculate an error function measuring the distance between the desired fixed output (target) and their own output, and adjust the connection strengths during the training process to minimize the result of the error function. Several models of supervised ANNs have been used, differentiated by topology and having their functioning dynamics managed by particular learning laws. In addition to the Back-Propagation network (BP), a typical Feed-Forward network trained with the Error Back-Propagation learning rule (or Delta Rule) (38), the Sine Net and BiModal learning laws were used [39,40].

The Sine Net (SN) is a family of multi-layer ANNs with a different unit activation function with respect to the BP. In the SN the input signal is non-linearly transformed through a non-monotonic sinusoidal function. A specific frequency and wave length in the sinusoidal function constrain the global input configuration and the connection weights, allowing a qualitative assessment of the output. The SN has excellent convergence and extrapolation capacities even on complex datasets.

The Bi-Modal networks (BM) are multi-layered networks that differ from BP and SN because all the hidden nodes are realized by 2 sub-nodes, each equipped with its own input connections: the first sub-node operates according to the descending gradient technique, the second through vectorial quantification. The outputs of the two nodes were then composed in a single output value. The Bi-Modal demonstrated an excellent convergence capacity on complex problems.

### Research protocols

The database was analyzed using two different research protocols: Random and Optimized.

In the Random Protocol [41], the global database was randomly split in two independent sub-samples. The two sets of independent sub-samples obtained were used, first as a training and testing set during the ANNs learning process and then were exchanged during the validation phase.

The Optimized Protocol also subdivided the entire database in two subsets of data but, in this case, the splitting was "optimized" by a data pre-processing able to extract training and testing (T&T) samples in which the case distribution represented the global structure of the database. The optimization procedure enhanced the reliability of the network model.

T&T was a system that performs an optimization of training and testing sub-samples through the iteration of data-splitting procedures on the global database. In the T&T system, based on the evolutionary algorithm GenD [42], the optimization algorithm distributed the original sample into two or more sub-samples with the aim of obtaining the maximum performance possible from an ANN model trained on the first sample and validated on the second. The T&T system was supported by the Input Selection (IS) [42] system, a variable selection technique based on the evolutionary algorithm GenD. When the database was built, the data were collected including all variables that may have had a correlation with the event being studied. The result of this approach was that a series of variables which did not contain information regarding the process being examined were present. These variables, inserted into the model, caused an increase of noise and a greater difficulty for the ANN to learn the data correctly. The IS system operated on a population of ANNs, each of them extracting a different pool of independent variables from a fixed dataset. Through the GenD evolutionary algorithm, the different selections of variable, generated by each ANN, may change over time, generation after generation. When the evolutionary algorithm no longer improves, the process stops, and the best selection of input variables is chosen and employed on the testing subset. The evolutionary algorithm searched for the minimal number of input variables that provided the best testing performance of the ANN model.

### Principal component analysis and connectivity map

A new mapping method was used to find out connectivity traces among variables, using a mathematical approach based on an artificial adaptive system, to define the strength of the associations of each variable with all the others in any dataset (the Auto Contractive Map-AutoCM algorithm- Copyright Semeion Research Centre). After the training phase, the weights matrix of the AutoCM represents the warped landscape of the dataset. Subsequently, a simple filter to the weights matrix of the AutoCM system was applied to obtain a map of the main connections between the variables of the dataset and the basic semantic of their similarities, defined connectivity map as detailed in the paper by Buscema and Grossi [35].

### Ethical approval

Informed consent was obtained from the mothers as appropriate. The study was approved by the local Ethics Committee (University of Parma Medical School).

## Results

### Random protocol

The first analysis, using the random protocol, yielded results obtained by the FeedForward architecture based on three learning laws: Back Propagation (BP), SineNet (SN), BiModal (BM). The input vectors for the ANNs contained 16 variables. The two target conditions, FGR and AGA (controls), were distinguished, approximately in 75% and 85% of the cases, respectively, with few mistakes (Table 3). The BP and the SN ANN models had similar performances and predicted FGR or AGA with the greatest accuracy.

**Table 3:** Random Protocol – Summary of results obtained by the Bi-modal (Bm), Back propagation (Bp) and Sine net (Sn) ANN models.

**ANN Model**	**Target**		**Mean Accuracy %**		**Errors**
	**FGR (%)**	**AGA (%)**	**Arithmetic**	**Weighted**	
**BM**	75,00	84.62	79,81	80,00	5
**BP**	75,00	84.62	79,81	80,00	5
**SN**	75,00	76,92	75,96	76,00	6

The percentage rates of correct classification of FGR and AGA are shown in the testing phase, together with the arithmetic and weighted mean accuracy.

FGR: fetal growth restriction;

AGA: appropriate for gestational age.

BM Bimodal neural network

BP: Back propagation neural network

SN: Sine net neural network

### Optimized protocol

In the optimized protocol the global database was processed twice by the IS system, reversing the two T&T subsets. From the initial 16 variables the IS system selected 7 variables the first time and 9 variables the second time (Table 4). The results of this analysis are summarized in Table 5. Also in this case the BP and SN ANNs gave the greatest accuracy and least errors in predicting FGR and AGA. In general, the ANNs performances were enhanced when processing the variables selected by IS instead of all the variables of the database; the optimized procedure allowed to predict FGR in approximately 92% of the cases and AGA in 85% of the cases with a low number of errors.

**Table 4:** Input variables selected by the optimized (I.S.) system.

**Variables**	**IS 1° selection**	**IS 2° selection**
Chronological age of the mother at delivery	X	X
Sex of the newborn		
Gestational age	X	X
Other siblings	X	X
Number of other siblings		
Number of previous abortions		
Total placental protein content in lysates		
IGF-II concentration in placental lysates	X	X
IGFBP-2 concentration in placental lysates	X	X
IL-6 concentration in placental lysates		
TNF-α concentration in placental lysates		
IGF-I relative gene expression in placenta	X	X
IGF-II relative gene expression in placenta		X
IGFBP-1 relative gene expression in placenta		
IGFBP-2 relative gene expression in placenta	X	X
IL-6 relative gene expression in placenta		X

**Table 5:** Optimized Protocol – Summary of results obtained by each ANN model on the variables of the first (7 variables) and second (9 variables) selection using the IS system.

**ANN Model**	**Target**		**Mean Accuracy %**		**Errors**
	**FGR (%)**	**AGA (%)**	**Arithmetic**	**Weighted**	
**BM 7 VAR**.	91,67	84,62	88,14	88,00	3
**BM 9 VAR**.	91,67	76,92	84,29	84,00	4
**BP 7 VAR**.	91,67	84,62	88,14	88,00	3
**BP 9 VAR**.	91,67	84,62	88,14	88,00	3
**SN 7 VAR**.	75,00	92,31	83,65	84,00	4
**SN 9 VAR**.	75,00	84,62	79,81	80,00	5

FGR: fetal growth restriction;

AGA: appropriate for gestational age.

BM Bimodal neural network

BP: Back propagation neural network

SN: Sine net neural network

### Principal component analysis, and connectivity map

**Figure 1:**
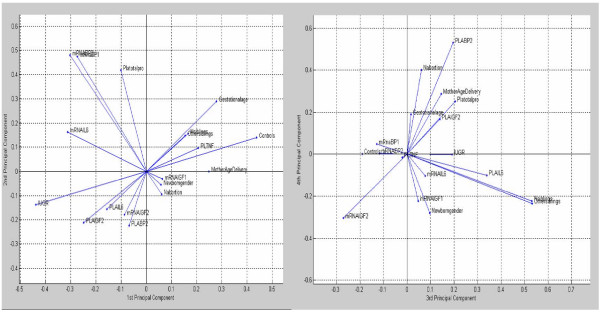
**Mapping of the first (left) and second (right) component analysis.** Four main clusters of variables emerged as described in results.

**Figure 2:**
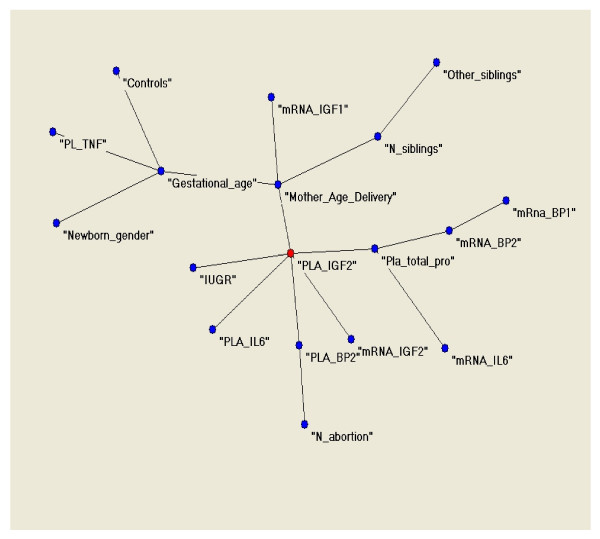
**Connectivity map clarifying the clusters of variables, and single relationships among variables.** Mother_Age_Delivery: Chronological age of the mother at delivery (years); newborn_gender: Sex of the newborn; Gestational_age: Gestational age (weeks); Other_siblings: Other siblings (yes or No); N_siblings: Number of other siblings; N_abortion: Number of previous abortions; Pla_total_pro: Total placental protein content in lysates (mg/ml); PLA_IGF2: IGF-II concentration in placental lysates (ng/mg); PLA_BP2: IGFBP-2 concentration in placental lysates (ng/mg); PLA_IL6: IL-6 concentration in placental lysates (pg/mg); PL_TNF: TNF- concentration in placental lysates (ng/mg); mRNA_IGF1:IGF-I relative gene expression in placenta (A.U./18S, UBQ); mRNA_IGF2:IGF-II relative gene expression in placenta (A.U./18S, UBQ); mRna_BP1: IGFBP-1 relative gene expression in placenta (A.U./18S, UBQ); mRNA_BP2:IGFBP-2 relative gene expression in placenta (A.U./18S, UBQ); mRNA_IL6:IL-6 relative gene expression in placenta (A.U./18S, UBQ); Controls: newborns appropriate for gestational age; IUGR: Intra uterine growth retardation (FGR fetal growth restricted).

The first and second principal components analysis are shown in Figure 1a. Different clusters of variables emerged (first cluster: FGR, IL-6, IGF-II and IGFBP-2 concentrations in placental lysates, and IGF-II mRNA; second cluster: total protein content in placental lysates, IGFBP-1, IGFBP-2 and IL-6 mRNAs; third cluster: IGF-I mRNA, newborn gender, number of abortions, mothers age at delivery; fourth cluster: gestational age, number and other siblings, TNF-α concentration in placental lysates and appropriateness for gestational ages). The third and fourth principal components gave clusters which were partially in contrast with the previous due to the limitations of the traditional PCA analysis (Figure 1b). However, the new algorithm provided a connectivity map which clarified the existent relationships at a higher level of analysis as represented in Figure 2.

IGF-II concentration in placental lysates resulted to be the principal hub of the system, i.e. the dominant variable. This variable was most tightly connected with the condition of being FGR at birth, with IGFBP-2 and IL-6 concentrations in placental lysates, IGF-II mRNA, and total placental protein concentration. This latter was related with placental mRNA for IGFBP-1, IGFBP-2 and IL-6. The concentration of IGFBP-2 in placental lysates was related with the number of spontaneous abortions.

Another close relationship was detected among the mother's age at delivery, gestational age and IGF-I mRNA in the placenta. There was also a relationship of the mother's age at delivery with the number of siblings. Gestational age was related with TNF-α concentration in placental lysates, with appropriateness for gestational age and newborn gender.

## Discussion

This study explored the association between the IGF and cytokine systems and fetal growth with complementary non-linear approaches: supervised neural networks (ANNS), and the semantic connectivity map.

Through ANNs, we established a consistent possibility to predict fetal growth status on the basis of selected variables with a low average error rate (8–15%). This meant that those variables contained specific information on the occurrence of FGR. In particular, IGF-II, IGFBP-2 and IL-6 concentrations in placental lysates were the most important determinants of fetal growth.

Through the connectivity map we established that IGF-II concentration in placental lysates was directly connected with the condition of FGR besides IL-6 and IGFBP-2 concentrations in lysates, and its corresponding mRNA.

A possible limit of the ANNS analysis is linked to the relatively small sample size and to the subsequent unbalanced ratio between variables and records. In this connection, however, adaptive learning algorithms of inference, based on the principle of a functional approximation as artificial neural networks, overcame the problem of dimensionality. The internal validation of the prediction accuracy is one of the most important problems in neural networks analysis. In fact, due to the restriction of training procedures to just a part of the data set, generally fifty percent, potential loss of power to recognize hidden patterns emerges.

A difficult problem was to establish the size and quality of the T&T sets because the complete data set was too small or too complex to be divided into two subsets on the basis of a pseudo-random criterion. However, the random division of a data set into two or three subsets was logical as we hypothesized that a simple function represented the data set in an optimal way. In this study the issue of optimization of the training and testing procedure was addressed with the use of the evolutionary 'T&T' algorithm, which ensured that the two halves of the data set contained the same amount of relevant information. Thus, the best division of the whole data set into a T&T set was reached after a finite number of generations.

The other major challenge in neural networks analysis on relatively small sample numbers in relation to a high number of variables, was the selection of the more predictive independent variables in order to reduce the so called "white noise" which would have limited the generalization capability of trained neural networks with respect to new records. In this study the issue of variables selection was addressed with the use of an evolutionary algorithm called IS system, which allowed the best approximation in a sustainable time of a typical non-polynomial hard problem which would have required an almost infinite computational time.

The principal component analysis has limits as shown by the different mapping obtained in the first and second versus the third and fourth component analysis. The use of Auto-CM, a new mapping system, overcomes these limits preserving non linear relationships, showing explicit connection schemes, capturing the complex dynamics of adaptive interactions and creates a "semantic connectivity map"[35].

Considering individual findings the connectivity map showed a relationship of total placental protein content with mRNA for different proteins which is easily explained as the amount of protein is dependent on the amount of mRNA translated into protein (relative gene expression). Previously, we had shown that there was no difference in the total amount of protein in the placenta of FGR and AGA newborns, which was confirmed [12].

The IGF-II peptide is recognized as an important determinant of fetal growth from animal [5] and human studies [8,10]. In placentas from FGR newborns, IGF-II is increased reflecting a possible protective mechanism to promote growth in unfavourable conditions [12]. The results of these analyses confirmed a major role of IGF-II for fetal growth. The connectivity map evidenced a direct relationship of IGF-II concentration in placental lysates and the condition of FGR, and suggested an effect of IL-6 and IGFBP-2 concentrations in the placenta on IGF-II. That IGF-II concentration in lysates was related with its corresponding mRNA was not surprising and confirms that one is looking at true biological effects.

This study did not confirm a major role for IGFBP-1 at variance with published experimental data [43,44]. We previously identified the lesser phosphorylated isoforms of IGFBP-1 to be the most increased in FGR; this finding suggested a protective mechanism in FGR which would be in line with the increased IGF-II concentrations observed in placental lysates [12]. This could explain the results of this study as only total IGFBP-1 concentration in the placenta was analysed which may not be the relevant factor.

IGFBP-2 concentration in placental lysates resulted to be an important variable in determining fetal growth. We previously described increased IGFBP-2 gene expression and peptide concentrations in the placenta in growth restricted fetuses, suggesting that IGF bioactivity might be significantly blunted in this condition [12]. The connectivity map did not show a direct relationship of IGFBP-2 with FGR suggesting an indirect effect possibly consisting in the regulation of IGF-II bioactivity.

The connection of IGFBP-2 concentration in lysates with the number of spontaneous abortions strengthens further the biological importance of this peptide in placenta for fetal growth, although we do not have an explanation for this.

IGFBP-2 has been poorly studied to date, and has not been considered previously an important bio-regulator of IGF bio-availability [11].

IL-6 has been studied only recently and very few data are available [13-15]. We showed that IL-6 mRNA was significantly increased in the placenta of FGR neonates [12]. The results of this analysis confirmed an important role for IL-6.

In contrast to IL-6, a significant role of TNF-α was not identified. Data in the literature in FGR are contrasting. Some studies report unchanged TNF-α mRNA expression in human placenta in FGR compared with controls [45] whereas others report increased TNF-α in the perfusate of FGR placentas [46]. TNF-α was increased in the serum and in the amniotic fluid of mothers with fetuses suffering of FGR [46,47]. We cannot exclude an indirect effect of TNF-α through the regulation of other yet unknown factors, however the connectivity map showed a connection only with gestational age. Gestational age was connected with appropriateness for gestational age also, possibly confirming that the more correct the duration of gestation the greater the possibility that a fetus is appropriate.

Finally, the connectivity map showed that the mothers age at delivery was connected with IGF-II concentration in lysates and IGF-I mRNA, possibly suggesting that maternal age is related with fetal growth. That maternal age is related with the number of siblings is a rule of human biology.

## Conclusion

These analyses identified the importance of the biochemical variables, IL-6, IGF-II and IGFBP-2 protein concentrations in placental lysates, and offered a new insight into placental markers of fetal growth within the IGF and cytokine systems, confirmed they had relationships and offered a critical assessment of studies performed to date. It remains to be elucidated whether the factors identified at the placental site are part of a pathological process or in connection with regulatory mechanisms induced by disturbances in the placenta resulting from previous pathological changes. The data provided useful information for the directions of future research, and the overall evidence suggested that further research in humans should focus on these biochemical data, at variance with the studies which have focused mainly on IGF-I and IGFBP-1.

The understanding of this system biology is relevant to the development of future therapeutical interventions possibly aiming at reducing IL-6 and IGFBP-2 concentrations preserving IGF bioactivity in both placenta and fetus.

## Competing interests 

The authors declare that they have no competing interests.

## Authors' contributions

MES: conceived and designed the study, supervised all the work related with the collection of the clinical data and all work in the laboratory, prepared the database for the specific analysis, interpreted the data, wrote the manuscript. EG: carried out the ANNS and PC analysis, was involved with the drafting of the related technical parts in the manuscript including tables and figures, and revised critically the entire manuscript. CV: helped supervise collection and analysis of clinical data, helped interpreting the data, writing and revising the manuscript. EF: helped collect biological and clinical data, interpret data and revise critically the manuscript. SB: acquired funding, supervised the research group, helped drafting and revising critically the entire manuscript. All authors read and approved the final manuscript.

## Pre-publication history

The pre-publication history for this paper can be accessed here:


